# The use of intonation for turn anticipation in observed conversations without visual signals as source of information

**DOI:** 10.3389/fpsyg.2015.00108

**Published:** 2015-02-10

**Authors:** Anne Keitel, Moritz M. Daum

**Affiliations:** ^1^Institute of Neuroscience and Psychology, University of Glasgow, Glasgow, UK; ^2^Research Group ‘Infant Cognition and Action’, Max Planck Institute for Human Cognitive and Brain Sciences, Leipzig, Germany; ^3^Department of Psychology, University of Zurich, Zurich, Switzerland

**Keywords:** turn-taking, conversations, intonation, visual cues, interaction, infants, adults, eye tracking

## Abstract

The anticipation of a speaker’s next turn is a key element of successful conversation. This can be achieved using a multitude of cues. In natural conversation, the most important cue for adults to anticipate the end of a turn (and therefore the beginning of the next turn) is the semantic and syntactic content. In addition, prosodic cues, such as intonation, or visual signals that occur before a speaker starts speaking (e.g., opening the mouth) help to identify the beginning and the end of a speaker’s turn. Early in life, prosodic cues seem to be more important than in adulthood. For example, it was previously shown that 3-year-old children anticipated more turns in observed conversations when intonation was available compared with when not, and this beneficial effect was present neither in younger children nor in adults (Keitel et al., 2013). In the present study, we investigated this effect in greater detail. Videos of conversations between puppets with either normal or flattened intonation were presented to children (1-year-olds and 3-year-olds) and adults. The use of puppets allowed the control of visual signals: the verbal signals (speech) started exactly at the same time as the visual signals (mouth opening). With respect to the children, our findings replicate the results of the previous study: 3-year-olds anticipated more turns with normal intonation than with flattened intonation, whereas 1-year-olds did not show this effect. In contrast to our previous findings, the adults showed the same intonation effect as the 3-year-olds. This suggests that adults’ cue use varies depending on the characteristics of a conversation. Our results further support the notion that the cues used to anticipate conversational turns differ in development.

## INTRODUCTION

Smooth and successful everyday social interactions are to a large extent based on the individual’s ability to anticipate what an interaction partner is going to do next. The anticipation of the next step is crucial for successful non-verbal interactions, for example, in sport or musical performances, but also for successful verbal interactions, that is, conversations. During a conversation, a variety of cues such as content or prosody mark the end of one speaker’s turn and the beginning of the next speaker’s turn. The present study was designed to explore the impact of two non-symbolic cues on the identification of a speaker’s turn in greater detail, with a particular focus on the development of this cue use: the prosodic cue intonation and visual signals that are unrelated to linguistic content and form.

When adults engage in a conversation, they identify turn transitions without great effort. They can use a variety of cues to do so: (1) the semantic content of a turn, or lexico-syntactic information, indicates that a response is required (*content cues*), (2) the spoken content is modulated by prosodic cues, such as intonation, to indicate a turn-boundary (*prosodic cues*), and (3) visual information peripherally accompanies speech, such as opening the mouth or gestures (*visual cues*). The use of lexico-syntactic content is highly related to language comprehension and seems to be among the most important factors for detecting the end of a turn. For example, de Ruiter et al. (2006) presented audio recordings of isolated turns from natural telephone conversations to adult participants, and asked them to indicate the anticipated end of the speaker’s turn. Participants reliably indicated an expected end of a turn well before the turn was actually finished. A follow-up study suggested that participants anticipated the upcoming lexical content and used this information to estimate the end of a turn (Magyari and de Ruiter, 2012). Accordingly, adults not only accurately detect a turn boundary, they usually anticipate it if they can rely on the spoken content.

Spoken language, however, not only includes semantic and syntactic cues but is also accompanied by rich non-symbolic information. Other linguistic information includes acoustically marked prosodic boundary cues (Gerken, 1996) that involve intonation, syllable length, and pauses. The availability of these cues helps to segment linguistic units even when information about content is unavailable (Schaffer, 1983; de Ruiter et al., 2006; Casillas and Frank, 2012). For example, when utterances are made unintelligible, with only prosodic cues (notably intonation) still intact, adults can identify the end and beginning of turns at above chance level (Schaffer, 1983; de Ruiter et al., 2006). In the reverse case, when utterances lack prosodic information, the ability to recognize the end of a turn is similar to when the full repertoire of information is available (de Ruiter et al., 2006). Thus, adults’ ability to detect an end of turn is not influenced by the availability of intonation (de Ruiter et al., 2006). These findings have led to the notion that adults primarily use prosody to predict the end of a turn if semantic and syntactic information is lacking (Grosjean and Hirt, 1996).

Visual information also contributes to turn taking in natural adult conversation. This includes visual cues such as mouth opening before speech onset, language-accompanying body movements and gesture, as well as gaze (Thórisson, 2002). For example, analyses of visual signals in conversations from a previous study by Keitel et al. (2013) yielded results indicating that the speakers opened their mouths on average 494.43 ms (SD = 228.48 ms) before a verbal speech signal was audible. Thus, it is well possible that visual cues support the detection of a turn. Taken together, adults make use of a variety of cues to detect turns during conversations, of which lexico-syntactic content seems to play a major role, and prosodic and visual information serve a supportive function.

Early in life, when language skills are far from adult level, children lack a substantial amount of the linguistic repertoire required for identifying the end of a turn. Studies on language development suggest that infants’ word comprehension starts at around the age of 8 months and rapidly increases over the next few months so that their productive vocabulary has reached approximately 600 words by the age of 30 months (Fenson et al., 1994). During this time, the child’s vocabulary not only increases substantially, but their sentences also become more complex (Clark, 2009). This development results in a rather sophisticated understanding and application of syntactic schemes at around age 3.5–4 years (Tomasello, 2000).

Regarding the anticipation of turns, eye tracking studies have shown that, with increasing language skills, children increasingly anticipate turns in observed conversations. One of the first studies that addressed this topic analyzed the gaze pattern of 1- and 3-year-old children during the observation of an everyday conversation between two speakers (von Hofsten et al., 2009). The authors of the study analyzed whether the observing children shifted their gaze to the next speaker before they started speaking. These results showed that the anticipation of turns increased significantly with age. Findings from similar eye tracking studies have shown that 1- to 7-year-old children anticipate speaker transitions in observed conversations effectively (Casillas and Frank, 2012, 2013). Furthermore, and most importantly for the present study, recent findings from Keitel et al. (2013) extended these results with a reliability analysis of participants’ gaze shifts. This analysis revealed that the anticipation of turns was reliable only in 3-year-olds and adults. Younger children shifted their gaze between speakers mostly independently of the turn-taking. These findings suggest that children need a sophisticated level of language understanding to anticipate conversations in a similar manner to adults, which is acquired around the age of 3 years.

Early in life, prosodic cues serve particularly important functions in children’s language development. For example, prosody helps 6-month-old infants to segment linguistic units, such as clauses (Nazzi et al., 2000) and phrases (Soderstrom et al., 2003). Furthermore, the prosodic cue intonation can already be extracted from speech by newborns (Nazzi et al., 1998; Sambeth et al., 2008). For the anticipation of turns in observed conversations, the role of intonation was investigated in the above-mentioned study by Keitel et al. (2013). In this study, children between 6 months and 3 years of age, as well as adults, saw videos of two dyadic conversations. The auditory signal of the conversations, in particular, intonation, was modulated. In one condition intonation was kept normal; in a second condition intonation was synthetically flattened. Interestingly, only the 3-year-old children benefitted from the unmodified and available intonation. Neither the adults nor the younger children showed differences in their gaze behavior between the two conditions. The lack of an intonation effect in younger children could be due to the fact that they often shifted their gaze between speakers unrelated to turn transitions. Nevertheless, intonation seems to be important for 3-year-olds to anticipate the course of observed conversations. However, a different study that investigated the role of lexical and prosodic information on 1- to 7-year-old children’s turn anticipation did not find this effect in 3-year-olds (Casillas and Frank, 2013). Casillas and Frank (2012, 2013) additionally differentiated between gaze shifts following questions or non-questions and found an advantage for questions in older children (beginning around 3–4 years). They conclude that children’s turn anticipation relies on both lexical and prosodic information. Thus, both studies, Keitel et al. (2013) and Casillas and Frank (2013), suggest that children use both lexical and prosodic cues to identify the end of turns. In contrast, studies with adults have concluded that they predominantly rely on the information provided by the lexical content (e.g., de Ruiter et al., 2006).

In the present study, we investigated the effect of the prosodic cue intonation in children and adults while controlling for visual cues (see also Casillas and Frank, 2013). During natural conversations between two human interaction partners, diverse visual cues can indicate an upcoming turn transition. For example, the mouth is usually opened before the actual stream of speech starts, and small gestures can also indicate a desire to respond. To avoid these visual cues entirely, we presented videos of conversations between two puppets. The puppets did not move their bodies, and the onset of the speech signal perfectly corresponded with the onset of the visual signal (mouth opening). The same was true for the offset of the acoustic and visual speech signals. Again, as in the study by Keitel et al. (2013), intonation was either normal or flattened. In addition to an adult control sample, we tested children aged 1 year, just starting their first words, and children aged 3 years, fluently using multi-phrasal utterances (von Hofsten et al., 2009; Keitel et al., 2013). The aim of the current study was to corroborate previous findings by Keitel et al. (2013) and von Hofsten et al. (2009) while exploring the impact of missing visual cues. Based on previous findings, we expected an increase of turn anticipations with age and a beneficial effect of available intonation at age 3.

## MATERIALS AND METHODS

### PARTICIPANTS

Seventy-two participants, 24 in each of the three age groups, were included in the final analyses: 1-year-old children [15 female, 9 male; *M*(age) = 12 months 4 days, range = 11 months 16 days to 12 months 15 days], 3-year-old children [13 female, 11 male; *M*(age) = 36 months 0 days, range = 35 months 17 days to 36 months 15 days], and adults [11 female, 13 male; *M*(age) = 23.5 years, range = 20–30 years]. Ten additional 1-year-olds and three additional 3-year-olds were tested but excluded from analysis because they were inattentive towards the conversations and did not yield enough valid trials (see Data Analysis for valid trials criteria). One additional adult participant was tested but was excluded from data analysis due to a technical error. Contact information of children was obtained from public birth records from the city of Leipzig, Germany. Children received a toy and adults received monetary compensation for their participation. The study was approved by a local ethics committee at the University of Leipzig and conducted in accordance with the Declaration of Helsinki.

### APPARATUS AND STIMULI

**FIGURE 1:**
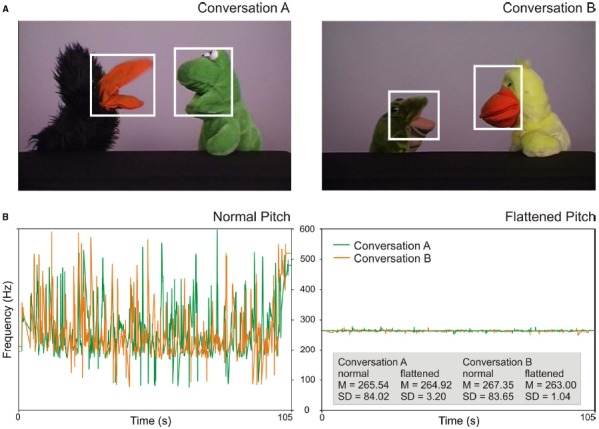
**(A)** Screenshots of the two presented conversations. The white boxes illustrate areas of interest for the analysis of gaze shifts towards speakers. **(B)** Pitch properties of the two conversations. Lines display the F0 contours of the normal conversations (left) and of the manipulated, flattened conversations (right). Mean pitch and standard deviations (in Hz) are indicated in the gray box.

Two different conversations between animal puppets were presented (see Figure 1A). The topics were recreational activities (conversation A) and birthday plans (conversation B). Each consisted of 28 turns that were analyzed (i.e., 27 speaker switches, or trials). The puppets first greeted the participants directly (these turns were not included in the analyses). The number of questions and declarative sentences were identical for each actor and conversation. The average length of speech and gaps differed only by approximately 295 (i.e., 11.9%) and 28 ms (3.1%), respectively, between conditions (see Table 1 for details of the conversations). The average duration of gaps in our study is longer than in a typical adult conversation (915 ms in our study vs. approximately 400–500 ms in other studies; see Heldner and Edlund, 2010). We decided to use distinct gaps to give even the younger children enough time to process them.

**Table 1:** **Details of the two conversations.** Number of analyzed speaker transitions, mean number of words per turn, total duration of conversations, mean duration of speech (i.e., mean duration of individual turn utterances), and mean duration of inter-turn gaps.

	**Number of analyzed speaker switches**	**Ø Words/ Turn**	**Duration (in seconds)**		
			**Total**	**Ø Speech**	**Ø Gaps**
Conversation A	27	7.9	88.24	2.34	0.93
Conversation B	27	9.3	95.44	2.63	0.90

To create the conversations, audio tracks were recorded first. Two female actors held the scripted conversations using normal, adult-directed intonation and spoke the lines into a microphone. Subsequently, the conversations were acted out using hand puppets that could open and close their mouths independently from the rest of the body. The audio recordings were played back during the video recording of the puppets so that the actors could move the puppet’s mouth synchronously with the speech signal. Audio and video tracks were then combined using video editing software (Edius). The tracks were arranged in a way that the movement of the mouth started and finished at exactly the same time as the speech. For both conversations, a second version was created with flattened intonation (using the same procedure as in Keitel et al., 2013). For the flattened intonation conversations, the variations of the fundamental frequency (F0) were removed and averaged to the mean frequency of the conversations using the software Praat (Boersma and Weenink, 2014). Via this procedure, the pitch contour of the conversations was extracted and segmented into pitch points at a rate of 100 Hz. The pitch points were removed, and a new pitch contour was created with the average frequency of the respective conversation using pitch synchronous overlap and add (PSOLA) resynthesis. This resulted in clearly less intonated, monotone speech (see Figure 1B for pitch properties). Thus, both conversations A and B were available in the normal condition and in the flattened condition.

The videos were presented on a 17-inch monitor (resolution: 800 × 600 pixels). They subtended a visual angle of 27.5° × 15.2°. The participants sat at a distance of approximately 60 cm from the monitor. Gaze was measured using a remote corneal reflection eye tracker (Tobii 1750, Stockholm, Sweden, with infant add-on; sampling rate: 50 Hz; precision: 1°; accuracy: 0.5°; software: ClearView 2.7.1). The stereo audio signal was played back via two speakers placed either side of the monitor.

### PROCEDURE

The experimenter explained the procedure to the children and their parents and to the adult participants; otherwise, participants were naïve to the purpose of the study. Written informed consent was obtained from the adult participants and from the children’s parents. After the child had become accustomed to the experimenter, the experiment started. Each participant was tested individually; one parent was present during testing. A 9-point infant calibration was used for all participants (this took approximately 30 s to 1 min) before the conversation videos were presented. Each participant watched both conversations, A and B, one with normal intonation and one with flattened intonation (this took approximately 3.5 min). The order of the conversations and the intonation were counterbalanced across participants. Before each video presentation, an attention-grabbing video (including interesting toys that moved and made sounds) was shown to the participants to focus their attention on the monitor.

### DATA ANALYSIS

Eye movement data were analyzed using the software Matlab R2013b (The MathWorks). To detect gaze shifts towards speakers, areas of interest (AOIs) were defined that covered each puppet’s mouth and eyes (see white boxes in Figure 1A). For the two speakers in conversation A, AOIs covered an area of 5.8° × 6.1°, and 6.0° × 7.2° visual angle, respectively. In conversation B, AOIs covered an area of 5.1° × 5.5°, and 4.9° × 6.5° visual angle, respectively.

Three measures were calculated to characterize participants’ gaze behavior. First, the exact time that gaze arrived at the next speaker relative to the beginning of their turn was calculated (*gaze latency*). A gaze shift towards the next speaker was considered to be anticipatory when it had arrived at the speaker before they had begun to speak, and reactive when it arrived after they had started speaking. Positive values indicate an anticipatory gaze shift; negative values indicate a reactive gaze shift. Second, we analyzed the location and duration of fixations provided by the data acquisition software (*fixation duration*; Keitel et al., 2014). Fixation duration can indicate distraction. For example, shorter fixation durations in the flattened condition could suggest that participants were distracted by the unusual intonation. Third, we calculated the occurrences of anticipatory and random gaze shifts (*occurrence rate*; Keitel et al., 2013). Occurrence rates are calculated as the number of gaze shifts during specific time intervals. For anticipatory gaze shifts, these time intervals were inter-turn gaps, including the final 500 ms prior to the end of a turn. The direction of anticipatory gaze shifts was always towards the (upcoming) speaker. For random gaze shifts, time intervals included speech (minus the 500-ms gap at the end of a turn). The direction of random gaze shifts was always away from the speaker (see Keitel et al., 2013 for detailed illustration of time intervals and direction of gaze shifts). Because occurrence rates are calculated as the number of occurrences per time interval, they can be interpreted as a probability to make a gaze shift. If the probability to make anticipatory gaze shifts is statistically higher than the probability to make random gaze shifts, turn anticipation is considered reliable.

Gaze shifts towards a speaker were only regarded valid if they were immediately preceded by a 100-ms fixation on the other speaker. This limitation was included to ensure that a gaze shift was related to the conversation and not random (Keitel et al., 2013, 2014, see also Melzer et al., 2012).

To be included in the analyses, a participant had to show at least 10 turn-taking-related gaze shifts (i.e., either anticipatory or reactive) in each condition, out of the 27 possible trials. In the group of 1-year-olds, children showed an average of 18.42 (SD = 4.72; normal condition), and 19.29 (SD = 4.20; flattened condition) valid trials, respectively. The 3-year-old children showed an average of 20.88 (SD = 4.37; normal condition), and 21.46 (SD = 3.90; flattened condition) valid trials, respectively. Adults showed an average of 24.75 (SD = 2.38; normal condition), and 24.71 (SD = 2.35; flattened condition) valid trials, respectively. Paired *t*-tests with number of valid trials between conditions did not suggest a difference for any age group (all *p*s > 0.38, two-sided). The results presented below are the same even with a simpler inclusion criterion—gazing at the screen at least 50% of the time in total—so it is unlikely that our inclusion criterion of 10 turn-taking-related gaze shifts introduced bias to our findings.

## RESULTS

### GAZE LATENCY

**FIGURE 2:**
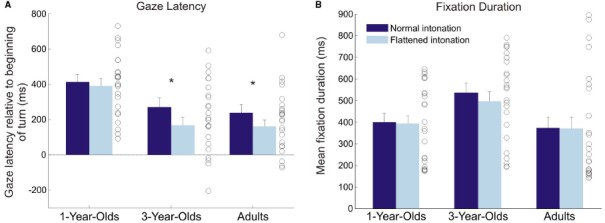
**Average gaze latency. (A)** and fixation duration **(B)** in the normal and flattened condition for all age groups. Error bars show standard error of the mean. Circles illustrate individual values for each participant averaged over both conditions. Asterisks indicate significant difference between conditions. Time point zero on the y-axis in the left plot refers to the beginning of a turn in the video. Positive values indicate that gaze arrived at the speaker before they started speaking; negative values indicate that gaze arrived after they had started speaking.

Initial analyses did not suggest any main effect or interaction effects of video order on gaze latency (all *p*s > 0.31), and data were collapsed over this factor. In all age groups and conditions, participants showed positive mean gaze latencies, which means, on average, they anticipated turns (*t*-tests against zero; 1-year-olds: normal condition: *t*(23) = 9.40, *p* < 0.001; flattened condition: *t*(23) = 9.37, *p* < 0.001; 3-year-olds: normal condition: *t*(23) = 5.53, *p* < 0.001; flattened condition: *t*(23) = 3.98, *p* = 0.001; adults: normal condition: *t*(23) = 5.32, *p* < 0.001; flattened condition: *t*(23) = 4.79, *p* < 0.001; see Figure 2A).

A 3 × 2 (age [1 year, 3 years, adults]) × condition [normal, flattened]) analysis of variance (ANOVA) with gaze latency yielded significant main effects of age, *F*(2,69) = 9.52, *p* < 0.001, $\eta_{G}^{2}$ = 0.17, and condition, *F*(1,69) = 7.33, *p* = 0.009, $\eta_{G}^{2}$ = 0.02, and no significant interaction, *F* < 1 (generalized eta-squared values are given to facilitate comparability with other studies, see Olejnik and Algina, 2000; Bakeman, 2005). Bonferroni-corrected *post hoc t*-tests showed that the 1-year-olds shifted their gaze earlier than the 3-year-olds, *p* = 0.001, and earlier than adults, *p* = 0.001. There was no significant difference between the gaze latencies of the 3-year-olds and the adults, *p* = 1. Following up the effect of condition, paired *t*-tests showed that 3-year-olds, *t*(23) = 2.30, *p* = 0.03, *d* = 0.47, as well as adults, *t*(23) = 2.17, *p* = 0.04, *d* = 0.44, displayed earlier gaze shifts in the normal than in the flattened condition, whereas 1-year-olds did not show this effect, *t*(23) = 0.47, *p* = 0.64,* d* = 0.10.

### DISTRIBUTION AND DURATION OF FIXATIONS

**FIGURE 3:**
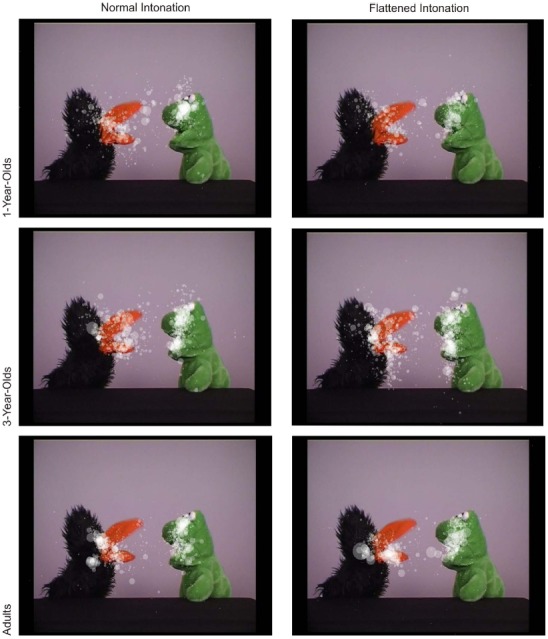
**Distribution of fixations in the normal and flattened condition (conversation A only) for all age groups.** Each transparent dot displays a fixation; its size indicates the fixation duration.

Figure 3 illustrates the fixation distribution in conversation A for both conditions (see Figure 2B for means of both conversations). The example illustrates similarly focused fixations on the puppets’ faces in both conditions. A 3 × 2 (age × condition) ANOVA with fixation duration yielded a significant main effect of age, *F*(2,69) = 3.26, *p* = 0.045, $\eta_{G}^{2}$ = 0.08, and no significant main effect of, or interaction effect with, intonation (both *F* < 1; see Figure 2B). Bonferroni-corrected *post hoc t*-tests showed that 3-year-olds had marginally longer fixation durations than adults, *p* = .06.

### OCCURRENCE RATE OF ANTICIPATORY AND RANDOM GAZE SHIFTS

**FIGURE 4:**
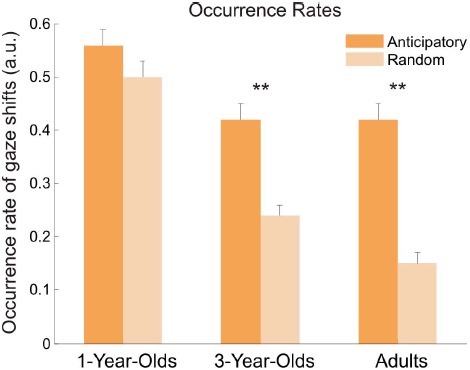
**Occurrence rates for anticipatory and random gaze shifts for all age groups.** Occurrence rates represent a probability to make a gaze shift to the other speaker. Error bars show standard error of the mean. Asterisks indicate significant difference between the rates for anticipatory and random gaze shifts.

The occurrence probabilities, or occurrence rates (see Figure 4), to make either anticipatory or random gaze shifts were entered into a 3 × 2 [age (1 year, 3 years, adults) × occurrences (anticipatory, random)] ANOVA. Results yielded main effects of age, *F*(2,69) = 31.17, *p* < 0.001, $\eta_{G}^{2}$ = 0.38, and occurrences, *F*(1,69) = 116.07, *p* < 0.001, $\eta_{G}^{2}$ = 0.20, and a significant interaction between both, *F*(2,69) = 15.06, *p* < 0.001, $\eta_{G}^{2}$ = 0.06. Bonferroni-corrected *post hoc t*-tests showed significant differences between 1-year-olds and both older age groups, both *p* < 0.001, but not between 3-year-olds and adults, *p* = 0.51. All age groups indicated larger occurrence rates for anticipatory gaze shifts than for random gaze shifts, but this difference was only significant in 3-year-olds, *t*(23) = 6.54, *p* < 0.001, *d* = 1.35, and adults,* t*(23) = 11.40, *p* < 0.001, *d* = 2.33. The 1-year-olds showed only marginally higher occurrence rates for anticipatory gaze shifts, *t*(23) = 1.92, *p* = 0.07, *d* = 0.39. Moreover, the rates for random gaze shifts decreased with age (all comparisons, *p* < 0.005); the rates for anticipatory gaze shifts differed only between 1-year-olds and older age groups, both *p* < 0.003, but not between 3-year-olds and adults, *p* = 0.92.

## DISCUSSION

### CHILDREN’S AND ADULTS’ CUE USE FOR TURN ANTICIPATION

In the present study we investigated the effect of intonation on turn anticipation during the observation of a dyadic conversation while controlling the availability of visual cues. The main finding was that 3-year-olds and adults anticipated more turns with normal intonation than with flattened intonation, whereas 1-year-olds did not show this effect. The effect of the children’s data replicates previous findings that 3-year-olds but not 1-year-olds benefit from the additionally available intonation (Keitel et al., 2013, but see Casillas and Frank, 2013). This was, as in the earlier study, not caused by general differences in the allocation of attention towards the conversation because fixation durations did not differ between conditions. In line with previous findings, we argue that at age 3, children have, on the one hand, learned to use prosodic boundary cues to indicate higher level linguistic aspects (Männel and Friederici, 2011) but, on the other hand, their overall language skills are not yet as sophisticated as in adults (e.g., Clark, 2009). Accordingly, at age 3, the information provided by intonation effectively supports the perception of conversations and helps to anticipate a speaker’s next turn.

The availability of the prosodic cue intonation yielded an increase of turn anticipations also in the adult participants. Usually, during the observation of normal conversations, adults heavily rely on lexico-syntactic cues (de Ruiter et al., 2006; Magyari and de Ruiter, 2012) and they only make use of prosodic cues when lexico-syntactic information is lacking (Grosjean and Hirt, 1996). The present findings, however, indicate that adults’ cue use is even more flexible: prosodic cues might be beneficial not only with lacking lexico-syntactic cues but also with lacking visual cues. This also gives rise to the assumption that adults naturally use visual cues in conversations to detect a speaker’s intention to respond. However, the lack of visual cues did not have a drastic effect on participants’ turn anticipation, as, on average, they anticipated an upcoming turn in both conditions. This supports at least the assumption that visual cues are not mandatory for turn anticipation.

### EXCEPTIONAL TURN ANTICIPATION IN 1-YEAR-OLDS

**FIGURE 5:**
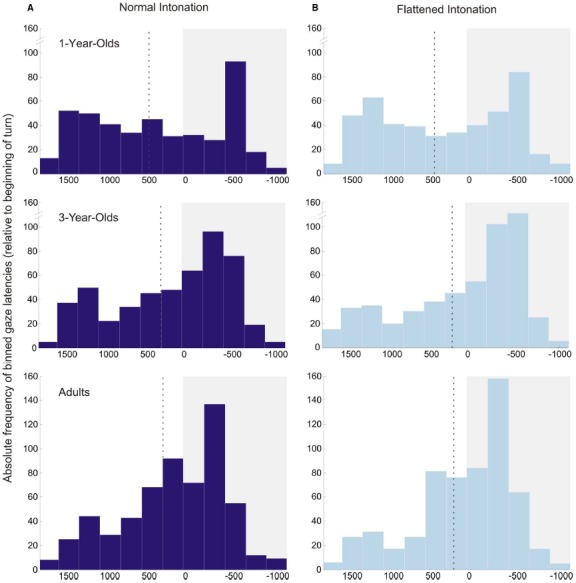
**Histograms of anticipatory and reactive gaze shifts in the normal (A) and flattened (B) condition for all age groups.** Values during a time interval with a white background illustrate anticipatory gaze shifts; values during a time interval with a gray background illustrate reactive gaze shifts (zero indicates beginning of turn). The dotted line in each plot displays the average gaze latency in this age group/condition. Bin size is 250 ms.

A second, somewhat unexpected, finding was that 1-year-olds anticipated more turns than older participants, independent of condition. The distribution of individual means in Figure 2 illustrates that these results were not caused by outliers, but that 1-year-olds were consistently good at anticipating the course of the conversations. However, the analysis of occurrence rates for anticipatory and random gaze shifts helps to interpret this finding: 1-year-olds generally showed a higher probability for making gaze shifts than 3-year-olds and adults. Importantly, there was no significant difference between the probabilities for making random and anticipatory gaze shifts, which suggests that 1-year-olds’ turn anticipation was not yet reliable. Furthermore, the probability of making random gaze shifts decreased significantly with age. This finding suggests that younger children gazed back and forth between the speakers much more often than older children and adults. The histograms of gaze latencies (Figure 5) illustrate that older participants’ gaze shifts to the next speaker center around the turn onset, whereas 1-year-olds’ gaze shifts to the next speaker are more evenly distributed over the whole time interval. An appropriate interpretation of these findings is that 1-year-olds shifted their gaze back and forth between speakers for the whole duration of the conversation, and this resulted in high probabilities of random *and* anticipatory gaze shifts. These constant gaze shifts could, on the one hand, be due to shorter attention spans in young children compared to older children and adults. On the other hand, puppets could have been particularly interesting for the 1-year-olds, resulting in keen visual exploration.

### ECOLOGICAL VALIDITY OF PUPPET CONVERSATIONS

The use of puppets as conversation partners in the present study raises the question of ecological validity and the tendency to generalize the present results to the “real world.” For human conversation partners, it is not possible to sit perfectly still or avoid mouth opening prior to speaking, without seeming unnatural or robotic. Therefore, the use of puppets to solve this problem seems justified (see also Casillas and Frank, 2013). Furthermore, studies have shown that young children and adults readily ascribe human qualities to non-human agents, even if they consist of geometrical shapes (Montgomery and Montgomery, 1999; Abell et al., 2000). Puppets should therefore make it easy for observers to immerse themselves in watching the conversations similar to a human conversation. This assertion is supported by the replication of results in 1- and 3-year-olds, compared with the findings of Keitel et al. (2013). However, a little uncertainty might remain that the adult findings are not due to the lack of visual signals but to the use of puppets. But even if this were the case, it would not affect the general conclusion of the present study that available information in a conversation results in differential cue use by adults. To partly resolve this issue, further studies could use puppets as conversation partners and include the typical lag between visual cues and the verbal speech signal.

## CONCLUSION

We investigated the effect of intonation on children’s and adults’ turn anticipation during the observation of dyadic conversations between puppets. When visual cues were lacking, both adults and 3-year-olds benefitted from the availability of intonation. Considering that adults did not show an intonation effect when visual cues were available (Keitel et al., 2013), this suggests that their cue use is rather flexible, depending on available information. Our results demonstrate further developmental differences in the perception of conversations: one-year-olds showed generally more gaze shifts when observing conversations than 3-year-olds and adults, and did not yet anticipate turns reliably. One the one hand, this makes interpretations of their cue use for turn anticipation difficult. On the other hand, a more fine-grained investigation into 1-year-olds’ many “random” gaze shifts might lead to a better understanding of the factors that influence their perception of conversations.

### Conflict of Interest Statement

The authors declare that the research was conducted in the absence of any commercial or financial relationships that could be construed as a potential conflict of interest.

## References

[B1] Abell F. Happé F. Frith U. (2000). Do triangles play tricks? Attribution of mental states to animated shapes in normal and abnormal development. Cogn. Dev. 15, 1–16 10.1016/S0885-2014(00)00014-9

[B2] Bakeman R. (2005). Recommended effect size statistics for repeated measures designs. Behav. Res. Methods 37, 379–384. 10.3758/BF03192707 16405133

[B3] Boersma P. Weenink D. (2014). Praat: Doing Phonetics by Computer [Computer program], Version 5.1.32. Retrieved from http://www.praat.org/

[B4] Casillas M. Frank M. C. (2012). “Cues to turn boundary prediction in adults and preschoolers,” in Proceedings of SemDial 2012 (SeineDial): the 16th Workshop on the Semantics and Pragmatics of Dialogue, eds Brown-Schmidt S. Ginzburg J. Larsson S. (Paris: Université Paris-Diderot), 61–69.

[B5] Casillas M. Frank M. C. (2013). “The development of predictive processes in children’s discourse understanding,” in Proceedings of the 35th Annual Meeting of the Cognitive Science Society, eds Knauff M. Pauen M. Sebanz N. Wachsmuth I. (Austin, TX: Cognitive Society), 299–304.

[B6] Clark E. V. (2009). First Language Acquisition. Cambridge, MA: Cambridge University Press.

[B7] de Ruiter J.-P. Mitterer H. Enfield N. J. (2006). Projecting the end of a speaker’s turn: a cognitive cornerstone of conversation. Language 82, 515–535 10.1353/lan.2006.0130

[B8] Fenson L. Dale P. S. Reznick J. S. Bates E. Thal D. J. Pethick S. J. (1994). Variability in early communicative development. Monogr. Soc. Res. Child Dev. 59, i+iii–v+1–185 10.2307/1166093 7845413

[B9] Gerken L. (1996). Prosody’s role in language acquisition and adult parsing. J. Psycholinguist. Res. 25, 345–356. 10.1007/BF01708577 8667302

[B10] Grosjean F. Hirt C. (1996). Using prosody to predict the end of sentences in English and French: normal and brain-damaged subjects. Lang. Cogn. Process. 11, 107–134 10.1080/016909696387231

[B11] Heldner M. Edlund J. (2010). Pauses, gaps and overlaps in conversations. J. Phon. 38, 555–568 10.1016/j.wocn.2010.08.002

[B12] Keitel A. Prinz W. Daum M. M. (2014). Perception of individual and joint action in infants and adults. PLoS ONE 9:e107450. 10.1371/journal.pone.0107450 25202914 PMC4174902

[B13] Keitel A. Prinz W. Friederici A. F. von Hofsten C. Daum M. M. (2013). Perception of conversations—the importance of semantics and intonation in children’s development. J. Exp. Child Psychol. 16, 264–277. 10.1016/j.jecp.2013.06.005 23876388

[B14] Magyari L. de Ruiter J. P. (2012). Prediction of turn-ends based on anticipation of upcoming words. Front. Psychol. 3:376. 10.3389/fpsyg.2012.00376 23112776 PMC3483054

[B15] Männel C. Friederici A. D. (2011). Intonational phrase structure processing at different stages of syntax acquisition: ERP studies in 2-, 3-, and 6-year-old children. Dev. Sci. 14, 786–798. 10.1111/j.1467-7687.2010.01025.x 21676098

[B16] Melzer A. Prinz W. Daum M. M. (2012). Production and observation of contralateral reaching: a close link by 12 months of age. Infant Behav. Dev. 35, 570–579. 10.1016/j.infbeh.2012.05.003 22728337

[B17] Montgomery D. E. Montgomery D. A. (1999). The influence of movement and outcome on young children’s attributions of intention. Br. J. Dev. Psychol. 17, 245–261.

[B18] Nazzi T. Floccia C. Bertoncini J. (1998). Discrimination of pitch contours by neonates. Infant Behav. Dev. 21, 779–784 10.1016/S0163-6383(98)90044-3

[B19] Nazzi T. Nelson D. G. Jusczyk P. W. Jusczyk A. M. (2000). Six-month-olds’ detection of clauses embedded in continuous speech: effects of prosodic well-formedness. Infancy 1, 123–147 10.1207/S15327078IN0101_11 32680315

[B20] Olejnik S. Algina J. (2000). Measures of effect size for comparative studies: applications, interpretations, and limitations. Contemp. Educ. Psychol. 25, 241–286. 10.1006/ceps.2000.1040 10873373

[B21] Sambeth A. Ruohio K. Alku P. Fellman V. Huotilainen M. (2008). Sleeping newborns extract prosody from continuous speech. Clin. Neurophysiol. 119, 332–341. 10.1016/j.clinph.2007.09.144 18069059

[B22] Schaffer D. B. (1983). The role of intonation as a cue to turn taking in conversation. J. Phon. 11, 243–257.

[B23] Soderstrom M. Seidl A. Kemler Nelson D. G. Jusczyk P. W. (2003). The prosodic bootstrapping of phrases: evidence from prelinguistic infants. J. Mem. Lang. 49, 249–267 10.1016/S0749-596X(03)00024-X

[B24] Thórisson K. R. (2002). “Natural turn-raking needs no manual: computational theory and model, from perception to action,” in Multimodality in Language and Speech Systems, eds Granström B. House D. Karlsson I. (Netherlands: Springer), 173–207 Retrieved from http://link.springer.com/chapter/10.1007/978-94-017-2367-1_8

[B25] Tomasello M. (2000). Do young children have adult syntactic competence? Cognition 74, 209–253 10.1016/S0010-0277(99)00069-4 10640571

[B26] von Hofsten C. Uhlig H. Adell M. Kochukhova O. (2009). How children with autism look at events. Res. Autism Spectr. Disord. 3, 556–569 10.1016/j.rasd.2008.12.003

